# A novel hand-crafted with deep learning features based fusion model for COVID-19 diagnosis and classification using chest X-ray images

**DOI:** 10.1007/s40747-020-00216-6

**Published:** 2020-11-12

**Authors:** K. Shankar, Eswaran Perumal

**Affiliations:** Department of Computer Applications, Alagappa University, Karaikudi, India

**Keywords:** COVID-19, Convolutional neural network, Preprocessing, Feature extraction, Fusion model, Classification

## Abstract

COVID-19 pandemic is increasing in an exponential rate, with restricted accessibility of rapid test kits. So, the design and implementation of COVID-19 testing kits remain an open research problem. Several findings attained using radio-imaging approaches recommend that the images comprise important data related to coronaviruses. The application of recently developed artificial intelligence (AI) techniques, integrated with radiological imaging, is helpful in the precise diagnosis and classification of the disease. In this view, the current research paper presents a novel fusion model hand-crafted with deep learning features called FM-HCF-DLF model for diagnosis and classification of COVID-19. The proposed FM-HCF-DLF model comprises three major processes, namely Gaussian filtering-based preprocessing, FM for feature extraction and classification. FM model incorporates the fusion of handcrafted features with the help of local binary patterns (LBP) and deep learning (DL) features and it also utilizes convolutional neural network (CNN)-based Inception v3 technique. To further improve the performance of Inception v3 model, the learning rate scheduler using Adam optimizer is applied. At last, multilayer perceptron (MLP) is employed to carry out the classification process. The proposed FM-HCF-DLF model was experimentally validated using chest X-ray dataset. The experimental outcomes inferred that the proposed model yielded superior performance with maximum sensitivity of 93.61%, specificity of 94.56%, precision of 94.85%, accuracy of 94.08%, *F* score of 93.2% and kappa value of 93.5%.

## Introduction

Coronavirus belongs to a huge family of viruses, which generally cause mild-to-moderate upper-respiratory tract illness similar to cold, namely Middle East respiratory syndrome (MERS) and Severe Acute Respiratory Syndrome (SARS) [1]. These illnesses generally occur in a wide range of animal species; however, in diverse cases, they tend to mutate and infect human beings quickly and spread to other people in an easier way. By the end of 2019, coronavirus 2019 (COVID-19, acronym of COronaVIrus Disease 19) started infecting human beings. The first case was identified by December 2019 in Wuhan city, China which rapidly spread across the globe.

Till now, there is a rapid evolution observed in coronavirus from 28 January 2020. By 15 February 2020, there were around 4600 COVID-19 affected cases globally with 160 mortalities. As of 22 September 2020, the total number of cases diagnosed is 31 million with 976,201 deaths. Wuhan city in China was under quarantine from 23 January 2020, restricting the transportation inside and outside the city. Such primary measures were prolonged in the subsequent days, to nearby cities of Huanggang, Zhijiang, Chibi, Jingzhou and Ezhou. Likewise, additional restrictions and orders were implemented globally. Many COVID-19 cases were diagnosed in Europe and Italy became the new epicenter in the month of March 2020.

In 5 April 2020, nearly 15.9 thousand fatalities were recorded by the Italian government. Out of this number, 8900 patients were from Lombardia, 2100 patients were staying in the zone of Emilia Romagna and 1200 patients from Piedmont. The morality rate in Italy increased by 19 March 2020 surpassing China. In medical perspective, COVID-19 inflammation generates a bunch of incurable pneumonia with medical issues alike SARS-CoV. Generally, patients experience influenza-like signs, such as difficulty in breathing, dry cough, tiredness and fever. In serious cases where the person has comorbidities, i.e., affected by other diseases like blood pressure, diabetes or heart problems, pneumonia develops rapidly resulting in acute renal failure and finally death in worst cases. But several patients are diagnosed with COVID-19 without symptoms. In Vo’ Euganeo, 50 km west of Venice, the total population of the country was made to undergo pharyngeal swab test while 50–75% of the populations were tested positive in swab, yet remained asymptomatic.

At present, the best method to determine COVID-19 is to perform swab test and examine the biotic material collected from patients using real-time reverse transcriptase polymerase chain reaction (RT-PCR). However, it is a challenge that the swab test is taken only for those individuals with COVID-19 symptoms. The existing COVID-19 patients without symptoms could not be recognized, until they approach the hospitals. Though the disease can be diagnosed by polymerase chain reaction, COVID-19 patients who are infected with pneumonia can be diagnosed using chest X-rays and computed tomography (CT) images only. In one of the studies conducted recently, COVID-19 can be slightly identified by human eye too [2]. COVID-19 transmission rate is calculated on the basis of volume of affected patients who are consistently diagnosed with minimum false negatives. Additionally, a less false-positive rate is essential to ensure not to push the medical system to extreme ends, by unreasonably revealing patients to isolation. With suitable contamination controller, it is proved that the earlier discovery of diseases enables the execution of helpful care essentials to COVID-19 patients.

By the end of January 2020, China conducted a research upon COVID-19 in terms of medical and paramedical specifications. The research conveyed that the COVID-19 cases exhibited some abnormal behaviors in chest CT scan images. World Health Organization (WHO) issued some other diagnostic protocols. Diagnosis is performed by real-time reverse transcriptase polymerase chain reaction (rRT-PCR) examination on biotic samples collected from patients. The experiments can be conducted in blood samples and the results are mostly obtained in within limited hours or within a day. As demonstrated earlier, COVID-19 can be probably deducted well by radiological images. Therefore, in this research, the authors estimate the prospects for the deduction of COVID-19 disease directly from medical images and X-ray scans.

Machine learning (ML)-based applications are currently employed for automatic disease diagnosis in healthcare sector [3]. DL is one of the common research domains in AI which allows the creation of end-to-end technique to attain assured outcomes. This is done utilizing intake data without any manual feature extraction. DL method has been effectively used in a number of issues like lung segmentation, skin cancer classification, fundus image segmentation, brain disease classification, pneumonia detection from chest X-ray images, breast cancer detection, and arrhythmia detection. Coronavirus pandemic is quickly raising the need for knowledge in this domain. It has improved awareness and emphasized the need for automatic detection technique based on AI. It is a risky process to provide radiologists for all the hospitals because of the scanty skilled manpower. Thus, the modest, precise, and fast AI methods might be useful to overcome these issues and give support to patients in correct time [4–6].

This paper introduces an effective fusion model (FM), hand-crafted with deep learning features called FM-HCF-DLF model for diagnosis and classification of COVID-19. The proposed FM-HCF-DLF model comprises three major processes, namely Gaussian filtering (GF)-based preprocessing, FM for feature extraction and classification. FM model incorporates the fusion of handcrafted features (HCF) using local binary patterns (LBP), whereas deep learning features (DLF) utilize convolutional neural network (CNN)-based Inception v3 approach. To further improve the performance of Inception v3 model, a learning rate scheduler using Adam optimizer has been applied in the current study. Finally, multilayer perceptron (MLP)-based classification process was executed to classify COVID-19 into different sets of classes. The proposed FM-HCF-DLF model was experimentally validated using chest X-ray dataset and the experimental outcome defined the superior performance of the presented model.

## Related works

With the advancements in healthcare image processing methods, there is a drastic increase observed in prediction and diagnostic devices [7]. ML methods are broadly known as projected tools to improve the diagnostic and prediction processes of numerous diseases [8]. Though effective feature extraction methods [9] are required to attain efficient ML techniques, DL is an extensive method which is approved in healthcare image system, thanks to its automated extraction feature like ResNet. Yu et al. [10] utilized Conventional Neural Network for classification of COVID-19-affected patients using chest CT imaging. Nardelli et al. [11] employed 3-DCNN to distinguish the respiratory artery veins from chest CT imaging. Shin et al. [12] utilized deep CNN to categorize the interstitial lung disease from CT imaging.

Xie et al. [13] categorized benign (lesion less than 3 cm) and malignant (lesion more than 3 cm) tumors based on pulmonary nodule classification. The study [14] arranged the melanoma dermoscopy images by DL with outstanding accuracy. The authors [15] observed the respiratory fissure in CT with the help of supervised discriminative learning platform. Setio et al. [16] implied multi-view convolutional networks to detect the lung nodules in CT imaging. Xia et al. [17] suggested deep adversarial networks to achieve segmentation on stomach CT imaging. Pezeshk et al. [18] utilized 3-D CNN to diagnose the pulmonary nodules in chest CT images. Zreik et al. [19] used a classifier method for recurrent CNN in the classification of Coronary Artery Plaque and Stenosis in Coronary CT.

Bhandary et al. [20] recommended a method to diagnose other respiratory disorder with the help of DL platform. Gao et al. [21] employed 3D block-based residual deep learning framework to detect severe stages of tuberculosis in CT scan and lungs’ X-ray imaging. Singh et al. [22] introduced particle swarm optimization related to adaptive neuro-fuzzy inference system (ANFIS) to improve the rate of classification. Zeng et al. [23] applied gated bi-directional CNNs (GCNN) which can be used for classifying COVID-19-affected patients. Based on in-depth analysis, it is determined that DL technique might attain effective outcomes for COVID-19 disease classifier from lung CT imaging. But these outcomes can be enhanced further if effective feature methods like variants of ResNet are used. In addition to this, the DL approaches can be hyper-tuned by transfer learning. Thus, a new deep transfer learning (DTL) development, related to COVID-19-affected patient classifier technique, is the major motivation behind the current research work.

## The proposed FM-HCF-DLF model

**Fig. 1:**
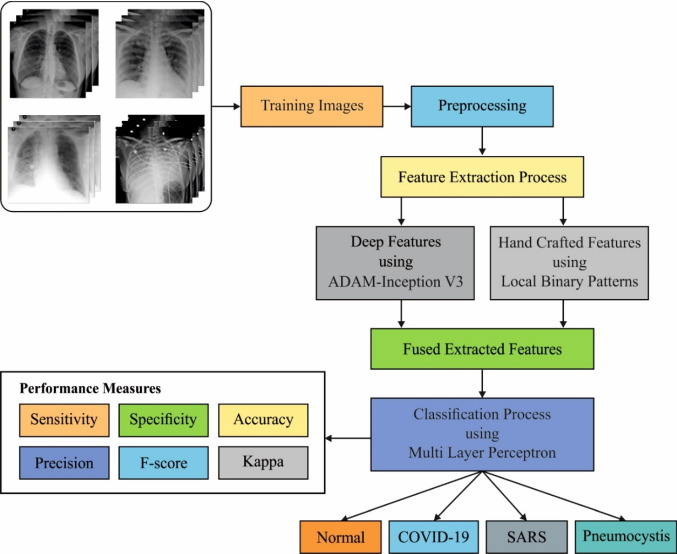
The working process of FM-HCF-DLF model

Figure 1 depicts the overall working principle of FM-HCF-DLF model. The figure states that the FM-HCF-DLF model involves preprocessing using GF technique to remove the noise that exists in the image. Then, FM-based feature extraction process takes place to extract the useful set of features from the preprocessed image. The HCF features use LBP whereas DLF uses CNN-based inception v3 model. Besides, Adam optimizer is utilized to adjust the learning rate of Inception v3 model. At last, MLP-based classification process is executed to identify and classify the chest X-ray images into different sets of classes.

### GF-based pre-processing

The execution of 2D Gaussian filter is employed extensively for smoothing and noise elimination. It needs massive processing resources whereas its efficiency in implementing is an inspiring research area. Convolution operators are defined as Gaussian operators and Gaussian smoothing is suggested by convolution. 1-D Gaussian operator is provided herewith

1 $${G}_{1D}\left(x\right)=\frac{1}{\sqrt{2\pi }\sigma }{e}^{-\left(\frac{{x}^{2}}{2{\sigma }^{2}}\right)}.$$

The best smoothing filter for images undergoes localization in spatial and frequency domains, where the uncertainty relation is satisfied as cited in the literature [24]:

2 $$\Delta x\Delta \omega \ge \frac{1}{2}.$$

2D Gaussian operator is demonstrated as follows:

3 $${G}_{2D}\left(x,y\right)=\frac{1}{2\pi {\sigma }^{2}}{e}^{-\left(\frac{{x}^{2}+{y}^{2}}{2{\sigma }^{2}}\right)},$$

where *σ* (Sigma) is the SD of a Gaussian function. When it has the maximum value, the image smoothing would be greater. $(x, y)$ denotes the Cartesian coordinates of the image that showcases the dimensions of window.

### Fusion-based feature extraction model

FM model incorporates the fusion of HCF using LBP and DLF with the help of Inception v3 technique. To further improve the performance of Inception v3 model, the learning rate scheduler is applied using Adam optimizer.

#### LBP features

LBP model is used in various domains and medical image analysis [25]. In LBP, the histograms are integrated as an individual vector where each vector is called as a pattern vector. Alternatively, the integration of LBP texture features and self-organizing map (SOM) is employed to find the effectiveness of the model. LBP is named as operator for texture definition based on differential symptoms over neighbor and central pixels. For all pixel values in the image, a binary code is obtained using thresholding of neighborhood with the help of middle pixel. The binary code is said to be a binary pattern. Therefore, the neighbor pixel is 1 when the pixel value is maximum than the threshold value. It becomes 0 when the pixel value is minimum than the threshold value. Following that, the histogram is deployed to calculate the frequency measured for binary pattern and every pattern denotes the possibility of binary pattern in an image.

The basic module of LBP operator utilizes the value of intermediate pixel as a threshold to $3\times 3$ neighbour pixels. Threshold task is applicable to deploy a binary pattern which refers a texture feature. The LBP process is depicted as follows:

4 $$ {\text{LBP}}\left( {u_{c} ,~v_{c} } \right) = \mathop \sum \limits_{{n = 0}}^{7} 2^{n} g\left( {I_{n}  - I\left( {u_{c} ,~v_{c} } \right)} \right) $$

$\mathrm{LBP}\left({u}_{c}, {v}_{c}\right)$ shows the LBP value at middle pixel $\left({u}_{c}, {v}_{c}\right)$. ${I}_{n}$ and $I\left({u}_{c}, {v}_{c}\right)$ are the measures of neighboring and centre pixels and index $n$ defines the index of neighbour pixels. The function $g\left(u\right)$ may be 0 while $u<0$ and $g\left(u\right)=1$ if $u\ge 0$. The adjacent pixels might be $0$, if the scores are lower than the threshold value. On the contrary, it may be 1 if the neighbor pixels are maximum than threshold. LBP value is estimated by scalar multiplication between binary and weight matrices. At last, the multiplication results are utilized to depict the LBP value.

#### CNN-based inception v3 features with Adam optimizer

**Fig. 2:**
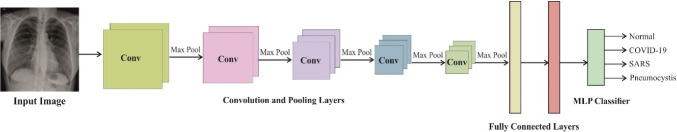
The structure of CNN

CNNs are enclosed with five layers, namely input, convolutional, pooled, FC, and output. GoogLeNet network is meant to be a CNN and is deployed in Google. It applies inception network method as it limits the number of network attributes and enhances the depth of a system. Therefore, it is extensively employed in image classifications. The instances of a general CNN are viewed as cited earlier [26] and are illustrated in Fig. 2.

##### Convolution layer

Convolution layer gets varied from a NN in which not all the pixels are linked to upcoming layer with a weight and bias. However, the whole image is divided into tiny regions after which weights and bias are used. Such weights and bias are named as filters or kernels that are convoluted with all small regions in the input image that offers a feature map. Such filters are referred to simple ‘features’ which can be explored from input image in this layer. The count of parameters is essential for this convolution task, which might be lower since a similar filter is traversed across the whole image for a single feature. The count of filters, size of local region, stride, and padding are referred to hyperparameters of convolution layer. According to size and genre of an input image, the hyperparameters undergo tuning to accomplish optimal outcomes.

##### Pooling layer

Pooling layer is applied to reduce the spatial dimensions of an image and the parameter count, and minimize the process. It performs a fixed function for an input without any parameters. Different types of pooling layers are available, such as average pooling, stochastic pooling, and max pooling. Max pooling is a common type and is applied in pooling algorithm, where $n\times n$ window is slid across and down the input with a stride of ‘s’. Every position of maximum value in $n\times n$ region is consumed and the input size becomes limited. It offers translational invariance where a small difference in a location would be applicable to analyze the image. Hence, the position is lost at the time of reducing the size.

##### Fully connected (FC) layer

Here, the flattened result of a last pooling layer is provided as input to FC layer. It acts as a CNN in which all the neurons of existing layer are linked to current layer. Thus, the count of parameters is maximum in the convolution layer. This FC layer is associated with an output layer named as classifier.

##### Activation function

Diverse activation functions are applied over different structures of CNN. Nonlinear activation functions have shown optimal outcome than former sigmoid or tangent functions. Such nonlinear functions are applied to enhance the training speed. Thus, various activation functions are applied and ReLU shows remarkable performance than alternate models.

CNN learning method relies upon vector calculus and chain rule. Assume $z$ to be a scalar (i.e., $z \in R$) and $y\in {R}^{H}$ as a vector, when $z$ is a function of $y$, the partial derivative of $z$, in terms of $y$, is a vector and can be determined as:

5 $${\left(\begin{array}{c}\partial z\\ \partial y\end{array}\right)}_{i}=\frac{\partial z}{\partial {y}_{i}}.$$

In particular, $$\left(\begin{array}{c}\partial z\\ \partial y\end{array}\right)$$ is a vector containing similar size as $y$, and its *i*th element is $${\left(\begin{array}{c}\partial z\\ \partial y\end{array}\right)}_{i}$$. And, it is noticeable that $$\left(\begin{array}{c}\partial z\\ \partial {y}^{T}\end{array}\right)={\left(\begin{array}{c}\partial z\\ \partial y\end{array}\right)}^{T}$$. In addition, assume $x\in {R}^{W}$ is another vector, and $y$ is a function of $x$. After that, the partial derivative of $y$ in terms of $x$ is determined by:

6 $${\left(\begin{array}{c}\partial z\\ \partial {y}^{T}\end{array}\right)}_{ij}=\frac{{\partial y}_{i}}{{\partial x}_{i}}.$$

In the fractional derivative $H\times W$ matrix, it is accessed at the juncture of *i*th row and *j*th column i.e., $\frac{{\partial y}_{i}}{{\partial x}_{i}}$. It looks simple to see that $z$ is a function of $x$ in a chain-like argument. Also, a function maps $x$ to $y$, and another function maps $y$ to $z$. The chain rule is utilized to compute as given herewith.

7 $$\left(\begin{array}{c}\partial z\\ \partial {x}^{T}\end{array}\right), \, {\text{as}} \, \left(\begin{array}{c}\partial z\\ \partial {x}^{T}\end{array}\right)=\left(\begin{array}{c}\partial z\\ \partial {y}^{T}\end{array}\right)\left(\begin{array}{c}\partial z\\ \partial {x}^{T}\end{array}\right).$$

The cost or loss function is utilized to measure the difference between the prediction of a CNN ${x}^{L}$ and the goal $t$, ${x}^{1}\to {w}^{1},{x}^{2}\to \ldots , {x}^{L}\to {w}^{L}=z$, utilizing a simplistic loss function $z={\Vert t-{x}^{L}\Vert }^{2}$. The predictive outcome is seen as ${\mathrm{argmax}}_{i} {x}_{i}^{L}$. A convolution method is represented as follows:

8 $${y}_{{i}^{l+1},{j}^{l+1},d}=\sum_{i=0}^{H}\sum_{j=0}^{W}\sum_{d=0}^{D}{f}_{i.j.d}\times {x}_{{i}^{l+1}+,i,{j}^{l+1}+j,d}^{L}.$$

Filter $f$ has size $(H\times W\times {D}^{l})$, so that the convolutional layer contains the spatial size of $\left({H}^{l}-H+1\right)\times ({W}^{l}- W+1)$ with $D$ slices which implies that $y \left({x}^{l+1}\right)$ in ${R}^{{H}^{l+1}\times {W}^{l+1}\times {D}^{l+1}}$, ${H}^{l+1}={H}^{l}-H+1$, ${W}^{l+1}={W}^{l}-W+1$ and ${D}^{l+1}=D$.

The possibility of all labels $k \in \left\{1,\ldots K\right\}$ is applied to train instance which is calculated by $P\left(k|x\right)=\frac{\mathrm{exp}({z}_{k})}{{\sum }_{i}^{K}\mathrm{exp}({z}_{i})}$, where $z$ is a non-normalized log possibility. A ground truth shared over labels $q(k|x)$ is normalized such that ${\sum }_{k}q(k|x)=1$. In this method, the loss is provided by cross-entropy and is defined below:

9 $$l=\sum _{k=1}^{K}\mathrm{log}\left(p\left(k\right)\right)q\left(k\right).$$

The cross-entropy loss is a differential value in terms of the logit ${z}_{k}$ and it is utilized in gradient training of deep methods since the gradient has the easier form $\frac{\partial l}{{\partial z}_{k}}=p\left(k\right)-q(k)$, bounded between − 1 and 1. Generally, if cross-entropy gets minimized, it implies that the log possibility of accurate label is maximized. Inception V3 is regarded as shared above labels which are independent of training instances $u(k)$ with a smooth parameter $\epsilon$, as a training instance, the label shared $q\left(k|x\right)={\delta }_{k,y}$ is easily returned by:

10 $${q}^{^{\prime}(k|x)}=\left(1-\epsilon \right){\delta }_{k,x}+\frac{\epsilon }{K}.$$

Otherwise, these are interpreted as cross-entropy as given below:

11 $$H\left({q}^{\prime},p\right)=-\sum_{k=1}^{K}\mathrm{log}\left(p\left(k\right)\right){q}^{{\prime}\left(k\right)}=\left(1-\epsilon \right)H\left({q}^{\prime},p\right)+\epsilon H\left(u,p\right).$$

So, the label-smoothing regularization is same for executing a single cross-entropy loss $H(q,p)$ and a couple of losses $H(q,p)$ and $H(u,p)$. Among these, the second loss penalizes the variation of the forecast label shared $p$ from prior $u$ with comparative weight $\frac{\epsilon }{(1-\epsilon )}$.

**Fig. 3:**
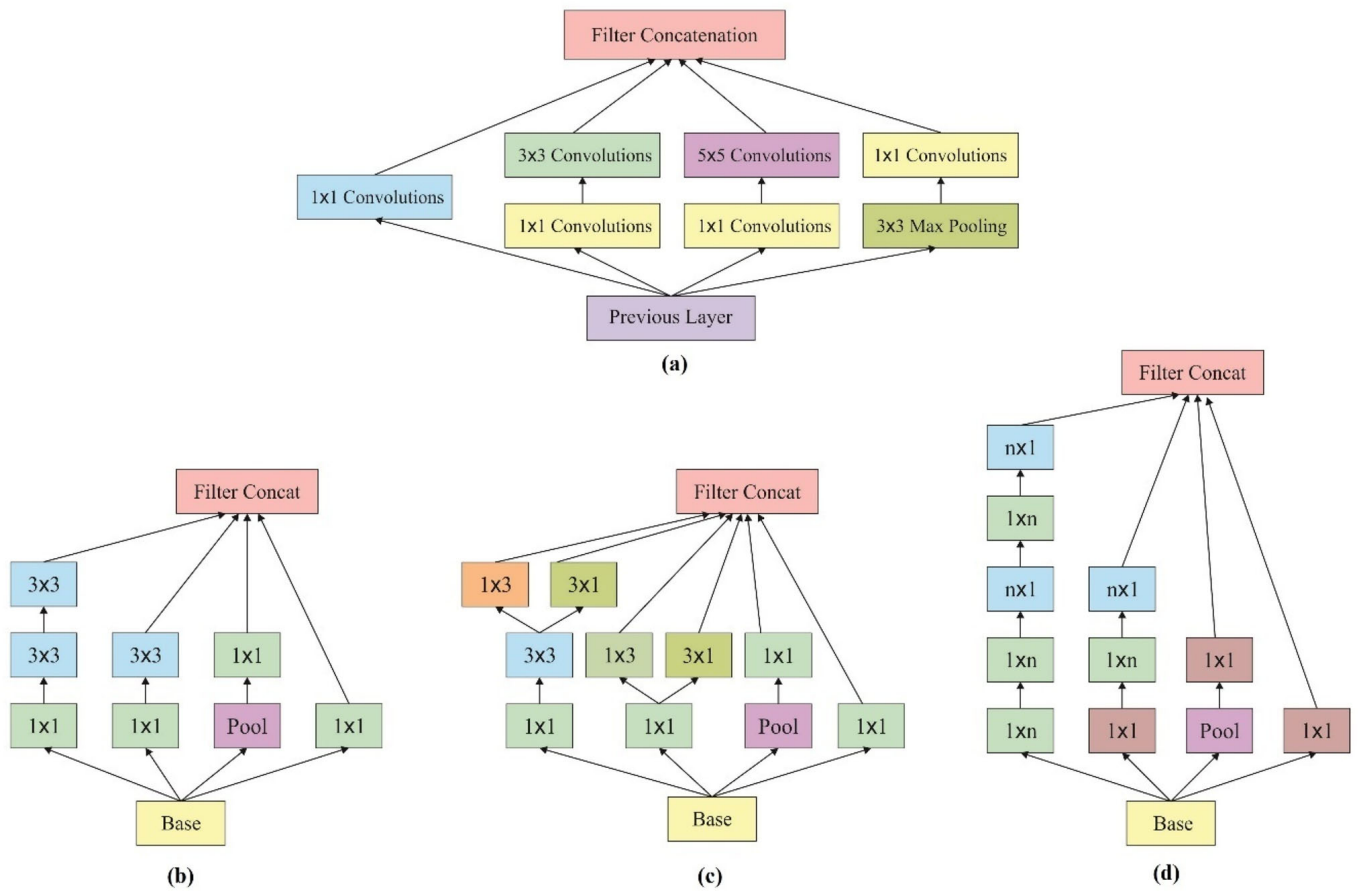
**a** Structure of inception model. **b**–**d** Inception V3 based inception module

The major objective of GoogLeNet network is to perform like an Inception network structure due to which the GoogLeNet method is named as Inception network [27]. It contains the maximum number of GoogLeNet versions which are classified into different versions, such as Inception v1, Inception v2, Inception v3, Inception v4, and Inception-ResNet. Thus, Inception generally includes three different sizes of convolution and maximum pooling. The result of network in previous layer is defined as the channel which is collected after the completion of convolution task and after nonlinear fusion is carried out. Similarly, the expression function of this network can be applied to various scales which can be enhanced while at the same time, the over-fitting problem can be eliminated. Figure 3a implies the structure of Inception network. Inception v3 refers a network structure deployed by Keras which is pre-trained in Image Net. The input size of the fundamental images is 299*299 with three channels. Also, Inception v3 network structure is applied in this study as shown in Fig. 3b. When compared to Inceptions v1 and v2, Inception v3 network structure employs a convolution kernel splitting model to divide massive volume integrals into minimum convolutions. For instance, a 3*3 convolution is divided into 3*1 and 1*3 convolutions. Using this splitting model, the count of attributes could be limited; thus, the network training speed can be enhanced at the time of extracting spatial feature in an effective manner. Simultaneously, Inception v3 optimizes the Inception network structure with the help of three different sized grids like 35*35, 17*17, and 8*8.

##### Learning rate scheduler

In DL training phase, it is suitable to limit a learning rate (${\gamma }_{t})$, when there is a progress development in training phase. The count of weights gets improved while training and this step is referred to step size or ‘learning rate’. Specifically, learning rate is an adjustable hyperparameter and is used for NN training using minimum positive values from 0.0 and 1.0. Additionally, learning rate balances the method of resolving the problems. Minimum learning rates require higher training epochs and they offer smaller alterations for weights whereas if the learning rates intends to offer enormous modifications, in such a case, it requires lower training epochs. The performance of tuning a learning rate is highly complex. The maximum learning rate results in divergent training process, while the minimum learning rate leads to slow convergence. An effective result can be accomplished by stimulating various learning rates at the time of training. The method applied for scheduling the leaning rate is named as ‘learning rate scheduler’. General learning rate schedules are different types, such as time-based decay, step decay as well as exponential decay.

Adam optimizer is an adaptive moment estimate optimizer which pursues a technique to 1st-order gradient-based optimizer. It depends on the adaptive estimation of lower-order moments. Here, ${g}_{t}$ represents the gradients, ${\theta }_{t}$ is the parameter at time $t$, ${\beta }_{1}$ and ${\beta }_{2}$ are assigned to be (0, 1), and $\alpha$ is the learning rate. Here, ${g}_{t}^{2}$ denotes the element-wise square of ${g}_{t} \odot {g}_{t}$ and the presented default settings are $\alpha$ = 0.001, ${\beta }_{1}$ = 0.9, ${\beta }_{2}$ = 0.999 and $\varepsilon ={10}^{-8}$. Every process on vector is element-wise defined, i.e., ${\beta }_{1}^{t}$ and ${\beta }_{2}^{t}$ in which ${\beta }_{1}$ and ${\beta }_{2}$ indicate to the power of $t$. The pseudocode for Adam technique is provided herewith. 
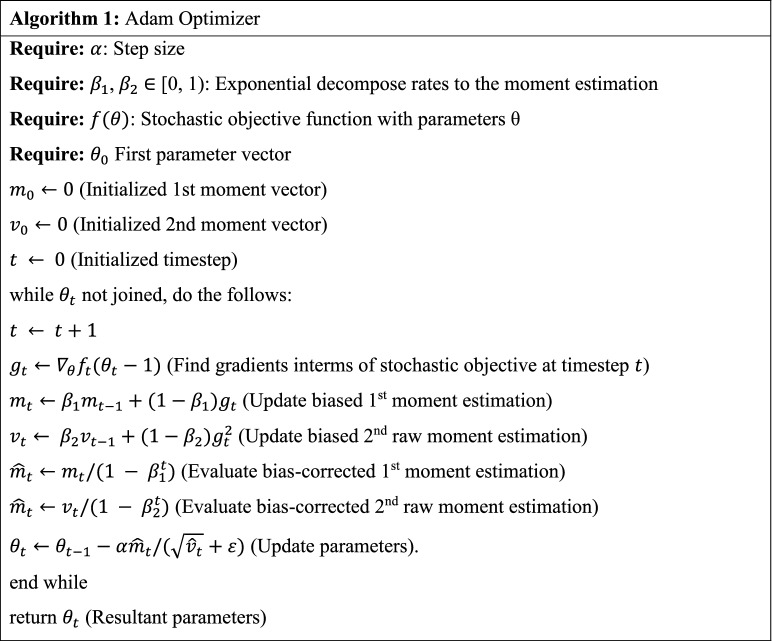


#### Fusion process

Data fusion has been employed in diverse ML and computer vision sectors. The features’ fusion is a significant operation that integrates a maximum number of feature vectors. The projected method depends upon features’ fusion by entropy. In addition, the obtained features are combined into single vector. There are three vectors computed herewith.

12 $$ \begin{aligned} {f}_{\mathrm{Inception}\_\mathrm{v}3\times m} & =\left\{{\mathrm{Inception}\_\mathrm{v}3}_{1\times 1},{\mathrm{Inception}\_\mathrm{v}3}_{1\times 2},\right. \\ &\quad \left. {\mathrm{Inception}\_\mathrm{v}3}_{1\times 3},\dots ,{\mathrm{Inception}\_\mathrm{v}3}_{1\times n}\right\},\end{aligned} $$

13 $${f}_{\mathrm{LBP}1\times p}=\left\{{\mathrm{LBP}}_{1\times 1},{\mathrm{LBP}}_{1\times 2},{\mathrm{LBP}}_{1\times 3},\dots ,{\mathrm{LBP}}_{1\times n}\right\}.$$

Then, the feature extraction is combined as a single vector.

14 $$\mathrm{Fused} \, {\left(\mathrm{feature} \, \mathrm{vector}\right)}_{1\times q}=\sum_{i=1}^{2}\left\{f{\mathrm{InceptionV}3}_{1\times m},f{\mathrm{LBP}}_{1\times p}\right\},$$

where $f$ implies a fused vector. The entropy is implemented on features’ vector for selected features only on the basis of a value given herewith.

15 $${B}_{\mathrm{He}}=-N{\mathrm{He}}_{b}\sum_{i=1}^{n}p\left({f}_{i}\right),$$

16 $${F}_{\mathrm{select}}={B}_{\mathrm{He}}\left(\mathrm{max}\left({f}_{i},1186\right)\right).$$

In Eqs. (15) and (16), $p$ denotes features’ probability and $He$ defines entropy. Finally, the selected features are offered to classification models so as to distinguish the X-rays from COVID.

### MLP-based classification

**Fig. 4:**
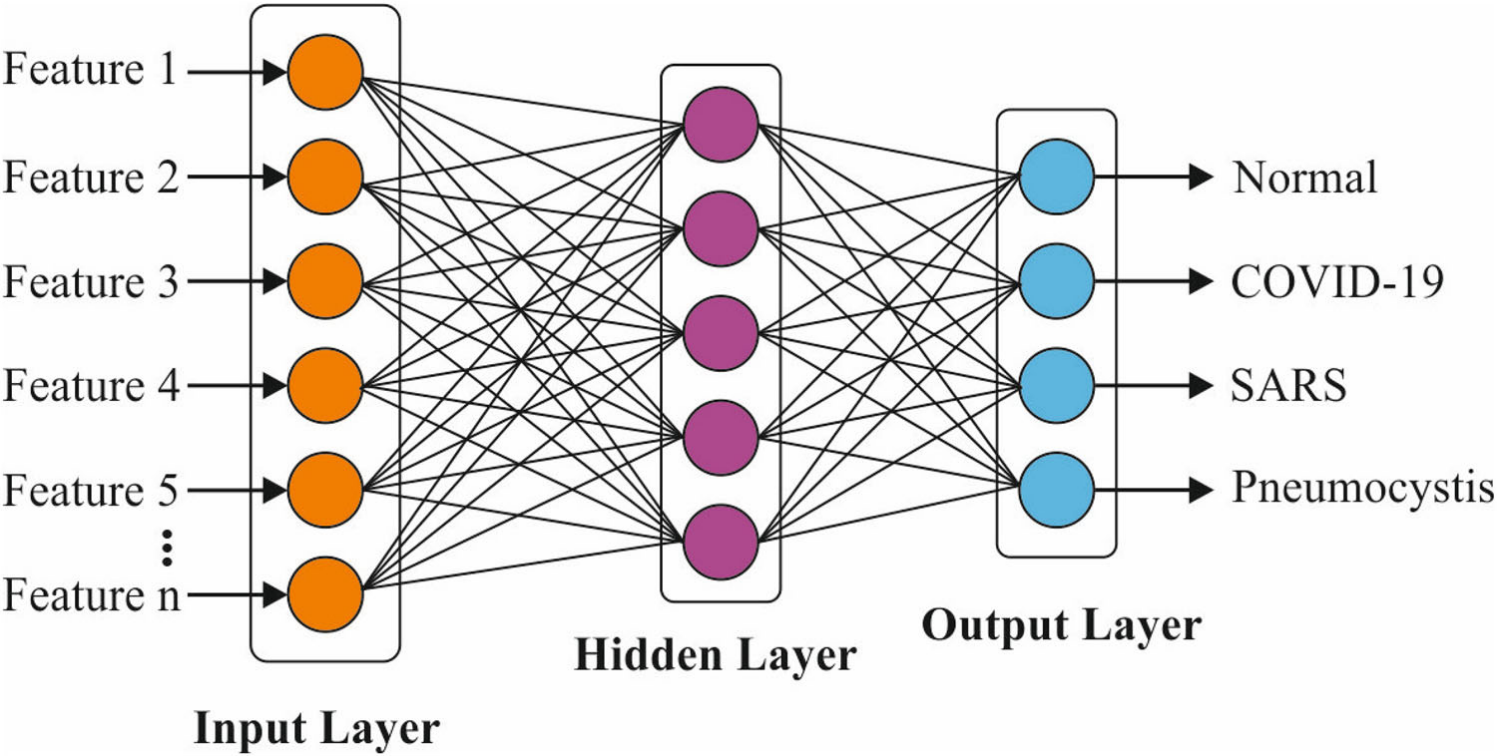
The structure of MLP

MLP network consists of three layers, namely input, hidden, and output layers. MLP network is capable of possessing numerous hidden layers. This is possible through the activation of network to hold processing abilities for the generation of system outputs. MLP is preferred over other classifiers due to the reasons listed herewith. MLP has adaptive learning process, i.e., capable of learning on how to perform tasks depending upon the training data. Besides, MLP does not require any consideration of the underlying probability density function. In addition, it offers the required decision function directly through training process. Figure 4 implies an MLP network with one hidden layer, which has few weights connecting among the layers. The final outcome scores are determined based on the given procedures. Initially, the addition of weights is estimated as following:

17 $${S}_{j}=\sum_{i=1}^{n}{w}_{ij}{x}_{i}+{\beta }_{i},$$

where *x*_*i*_ denotes an input variable, *w*_*ij*_ defines the weight between input variable *x*_*i*_ and neuron *j*, and β_*i*_ depicts the bias term of the input variable. Then, the final values of the neurons in hidden layers are produced from the obtained values of weighted summation (Eq. 17), by an activation function.

A well-known choice of these functions is said to be a sigmoid function as given herewith.

18 $${f}_{j}\left(x\right)=\frac{1}{1+{e}^{-{S}_{j}}},$$

where *f*_*j*_ represents the sigmoid function for neuron *j* and *S*_*j*_ refers to sum of weights. As a result, the result of neuron *j* is determined as following:

19 $${y}_{j}=\sum_{i=1}^{k}{w}_{ij}{f}_{j}+{\beta }_{j},$$

where *y*_*j*_ signifies the result of neuron *j*, *w*_*ij*_ denotes the weight from output variable *y*_*i*_ and neuron *j*, *f*_*j*_ indicates the activation function for neuron *j*, and β_*i*_ depicts the bias term of the final variable.

## Performance validation

The proposed MMF-DTL model was implemented in a PC with configurations, such as Intel i5 processor, 8th generation PC with 16 GB RAM, MSI L370 Apro, Nividia 1050 Ti4 GB. The authors used Python 3.6.5 tool along with pandas, sklearn, Keras, Matplotlib, TensorFlow, opencv, Pillow, seaborn and pycm. The parameter setting is given as follows: epoch count: 35, batch size: 4098, learning rate: 0.01, and beta: 0.9. A sample visualization of the processes involved in the experimentation is shown in the appendices 1, 2, 3 and 4.

### Dataset details

**Fig. 5:**
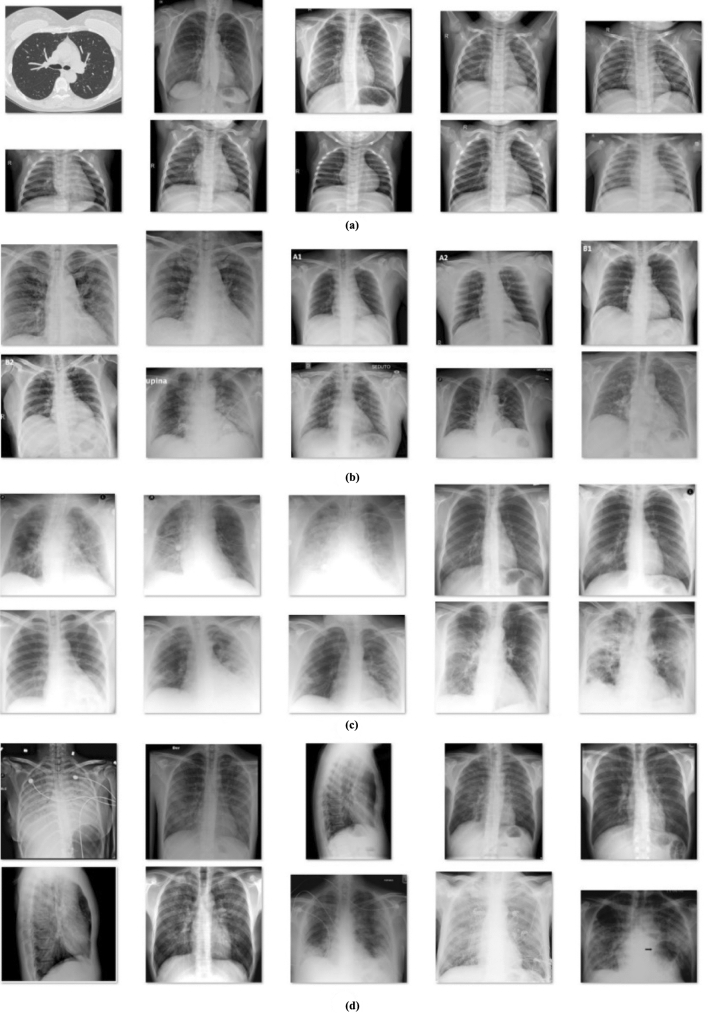
Sample test images: **a** normal, **b** COVID-19, **c** SARS, **d** pneumocystis

The proposed FM-HCF-DLF model was assessed for its performance using chest X-ray dataset [28]. The dataset is composed of 27 images under normal class, 220 images under COVID-19, 11 images under SARS and 15 images in Pneumocystis class. A sample set of images from the dataset is shown in Fig. 5. The authors used fivefold cross-validation.

### Results

**Fig. 6:**
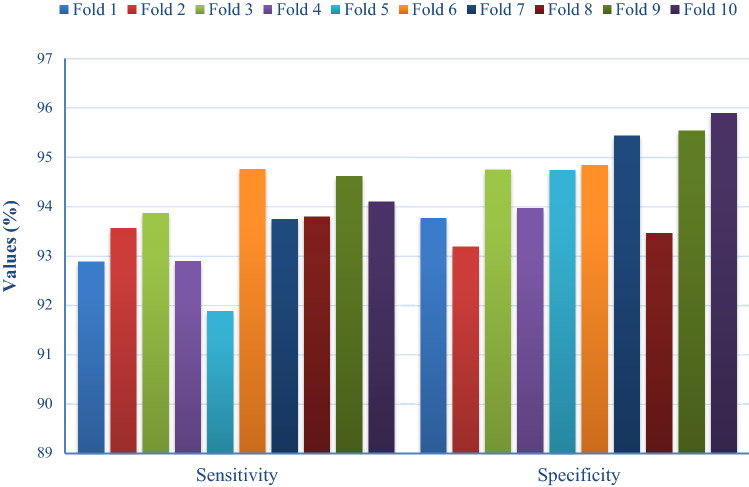
Sensitivity and specificity analysis of FM-HCF-DLF model in terms of different folds

**Fig. 7:**
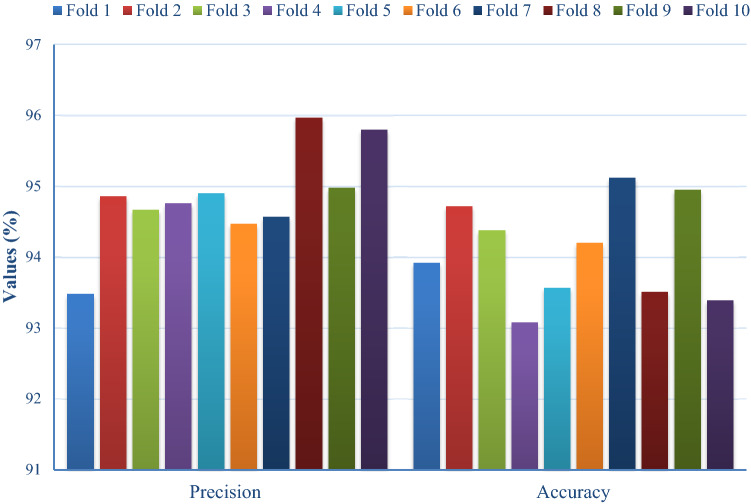
Precision and accuracy analysis of FM-HCF-DLF model in terms of different folds

**Fig. 8:**
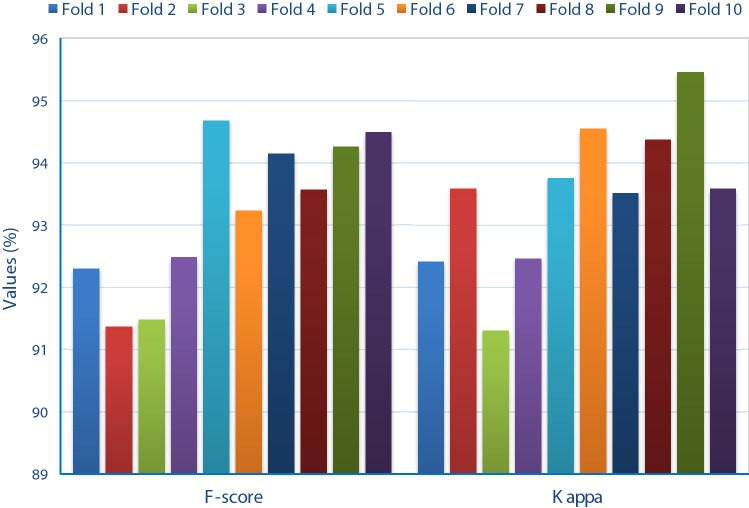
*F* score and kappa analysis of FM-HCF-DLF model in terms of different folds

**Fig. 9:**
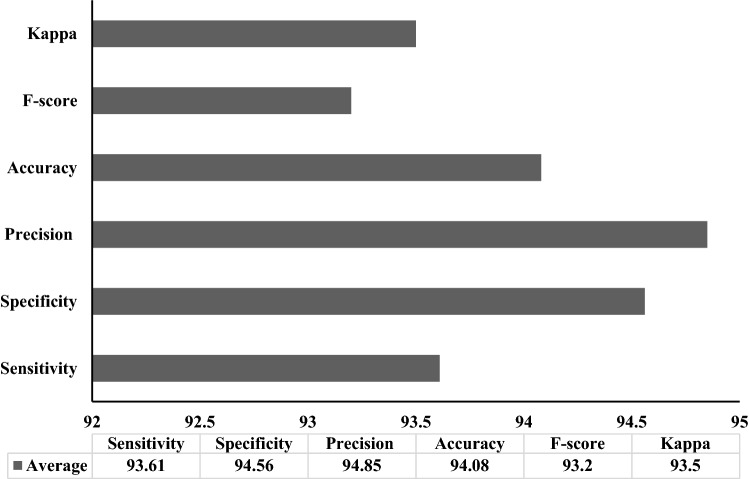
Average analysis of FM-HCF-DLF model in terms of different measures

Table 1 and Figs. 6, 7, 8, 9 show the results of analysis conducted upon FM-HCF-DLF model in terms of diverse measures under varying numbers of folds. Figure 6 shows the sensitivity and specificity analyses of FM-HCF-DLF model under varying fold counts. Under fold 1, the FM-HCF-DLF model attained the maximum sensitivity and specificity values of 92.89% and 93.77%, respectively. Similarly, under fold 2, the presented FM-HCF-DLF model resulted in higher sensitivity and specificity values being 93.56% and 93.87%, respectively. Likewise, under fold 3, the projected FM-HCF-DLF method yielded the maximum sensitivity and specificity values, such as 93.87% and 94.75%, correspondingly. Further, under fold 4, the presented FM-HCF-DLF approach accomplished greater sensitivity and specificity, i.e., 92.90% and 93.97%, respectively. Along with that, under fold 5, the implied FM-HCF-DLF scheme exhibited optimal sensitivity and specificity values of 91.88% and 94.74% correspondingly. Accordingly, under fold 6, the applied FM-HCF-DLF technique produced a better sensitivity and specificity of 94.76% and 94.84% correspondingly. Under fold 7, the newly developed FM-HCF-DLF method resulted in high sensitivity and specificity values, such as 93.74% and 95.44%, respectively. Further, under fold 8, the deployed FM-HCF-DLF technique implied the best sensitivity and specificity values of 93.80% and 95.44% correspondingly.

**Table 1:** Results of the analysis of proposed FM-HCF-DLF model in terms of different measures and folds

No. of folds	Sensitivity	Specificity	Precision	Accuracy	*F* score	Kappa
Fold 1	92.89	93.77	93.48	93.92	92.30	92.41
Fold 2	93.56	93.19	94.86	94.72	91.37	93.58
Fold 3	93.87	94.75	94.67	94.38	91.48	91.30
Fold 4	92.90	93.97	94.76	93.08	92.48	92.46
Fold 5	91.88	94.74	94.90	93.57	94.68	93.75
Fold 6	94.76	94.84	94.47	94.20	93.23	94.55
Fold 7	93.74	95.44	94.57	95.12	94.15	93.51
Fold 8	93.80	93.47	95.97	93.51	93.57	94.37
Fold 9	94.62	95.54	94.98	94.95	94.26	95.45
Fold 10	94.10	95.89	95.80	93.39	94.49	93.58
Average	93.61	94.56	94.85	94.08	93.20	93.50

In line with this, under fold 9, the applied FM-HCF-DLF method yielded a better sensitivity and specificity of 94.62% and 95.54%, respectively. Simultaneously, under fold 10, the applied FM-HCF-DLF model produced the maximum sensitivity and specificity values, such as 94.10% and 95.89%, respectively.

Figure 7 implies the precision and accuracy analyses of the FM-HCF-DLF model under diverse fold counts. Under fold 1, the FM-HCF-DLF approach accomplished better precision and accuracy values, such as 93.48% and 93.92%, correspondingly. Further, under fold 2, the proposed FM-HCF-DLF method accomplished the maximum precision and accuracy values of 94.86% and 94.72%, respectively. Under fold 3, the applied FM-HCF-DLF approach exhibited better precision and accuracy values, i.e., 94.67% and 94.38% correspondingly. In line with this, under fold 4, the proposed FM-HCF-DLF model achieved a greater precision and accuracy of 94.76% and 93.08%, respectively. Under fold 5, the developed FM-HCF-DLF model produced higher precision (94.90%) and accuracy (93.57%) values, respectively. In line with this, under fold 6, the implied FM-HCF-DLF model exhibited the maximum precision and accuracy values of 94.47% and 94.20%, respectively. Under fold 7, the deployed FM-HCF-DLF technique demonstrated excellent precision and accuracy values, such as 93.57% and 95.12%, correspondingly. When using fold 8, the projected FM-HCF-DLF technique attained a greater precision value of 95.97% with accuracy being 93.51%. Along with that, under the fold 9, the proposed FM-HCF-DLF approach attained the maximum precision values of 94.98% with 94.95% accuracy. In line with this, under fold 10, the deployed FM-HCF-DLF model resulted in optimal precision and accuracy values of 95.80% and 93.39%, respectively.

Figure 8 illustrates *F* score and kappa analyses of FM-HCF-DLF approach under different fold counts. Under fold 1, the FM-HCF-DLF technique achieved high values in *F* score and kappa, such as 92.30% and 92.41%, correspondingly. In line with this, under fold 2, the applied FM-HCF-DLF approach accomplished higher *F* score and kappa values, i.e., 91.37% and 93.58%, respectively. Likewise, under fold 3, the applied FM-HCF-DLF framework demonstrated the maximum *F* score and kappa values being 91.48% and 91.30% correspondingly. Further, under fold 4, the projected FM-HCF-DLF scheme accomplished high *F* score and kappa values, i.e., 92.48% and 92.46%, respectively. Simultaneously, under fold 5, the projected FM-HCF-DLF technology exhibited optimal *F* score and kappa values of 94.68% and 93.75%, respectively. In line with this, under fold 6, the applied FM-HCF-DLF model attained the optimal *F* score and kappa values, such as 93.23% and 94.55%, correspondingly. Under fold 7, the implied FM-HCF-DLF model secured optimal *F* score value, i.e., 94.15% and kappa value i.e., 93.51%. In fold 8, the provided FM-HCF-DLF technique depicted maximum *F* score and kappa values of 93.57% and 94.37%, respectively. The proposed FM-HCF-DLF approach yielded better *F* score and kappa values of 94.26% and 95.45% when applied under fold 9. In alignment with this, under fold 10, the deployed FM-HCF-DLF technology implied a high *F* score and kappa of 94.49% and 93.58%, respectively.

Figure 9 displays the average results of the analysis of FM-HCF-DLF model in terms of diverse measures. The figure points out that the FM-HCF-DLF model reached the maximum sensitivity of 93.61%, specificity of 94.56%, precision of 94.85%, accuracy of 94.08%, *F* score of 93.2% and kappa value of 93.5%.

**Fig. 10:**
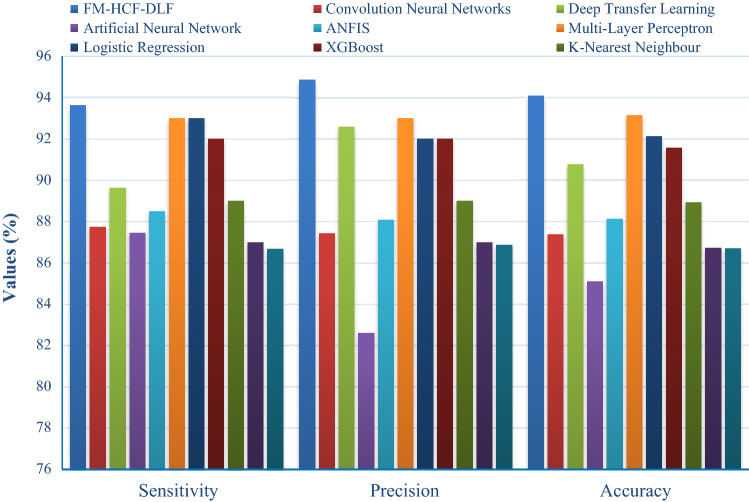
The comparative analysis of FM-HCF-DLF model with existing methods

Table 2 and Fig. 10 provided compares the results of the proposed model with that of other models, such as CNN, DTL, artificial neural network (ANN), ANFIS, MLP, logistic regression (LR), XGBoost, K-nearest neighbor (KNN), decision tree (DT) and Xiaowei Xu et al. [29] models. The table values indicate that the model devised by Xiaowei Xu et al. and DT achieved only minimal sensitivity values of 86.67% and 87%. Further, CNN and ANN models showed slightly better sensitivity values of 87.73% and 87.45%, respectively. Along with that, ANFIS and DTL models attained closer sensitivity values of 88.48% and 89.61%, respectively. At the same time, the XGBoost model resulted in a slightly higher sensitivity value of 92%. Besides, MLP and LR models yielded higher and identical sensitivity value, i.e. 93%. However, the proposed FM-HCF-DLF model achieved superior sensitivity value of 93.61%. The table values represent that the ANN method resulted in a minimum specificity of 82.91%. Similarly, the CNN approach accomplished a moderate specificity of 86.97. Likewise, the ANFIS model produced nearby specificity value, i.e., 87.74%. Simultaneously, the DTL method offered better specificity value of 92.03%. However, the presented FM-HCF-DLF model attained the best specificity value of 94.56%. The table values point out that the ANN method acquired the least precision and accuracy values, such as 82.59% and 85.09%, respectively.

**Table 2:** Result of the analysis of existing methods with proposed method

Models	Sensitivity	Specificity	Precision	Accuracy	*F* score
FM-HCF-DLF	93.61	94.56	94.85	94.08	93.20
CNN	87.73	86.97	87.41	87.36	–
DTL	89.61	92.03	92.59	90.75	–
ANN	87.45	82.91	82.59	85.09	–
ANFIS	88.48	87.74	88.08	88.11	–
MLP	93.00	–	93.00	93.13	93.00
LR	93.00	–	92.00	92.12	92.00
XGBoost	92.00	–	92.00	91.57	92.00
KNN	89.00	–	89.00	88.91	89.00
DT	87.00	–	87.00	86.71	87.00
Xiaowei Xu et al	86.67	–	86.86	86.70	86.70

Simultaneously, CNN and ANN methodologies implied considerable precision and accuracy values of 87.41% and 87.36% for former and 82.59% and 85.09% for latter methodologies. The model developed by Xiaowei Xu et al. and DT approach attained nearby precision and accuracy values, such as 86.86%, 86.70% and 87%, 86.71%, correspondingly. Following that, ANFIS and KNN frameworks offered moderate and closer precision and accuracy values, such as 88.08%, 88.11% for the former and 89%, 88.91%, for the latter, respectively. Similarly, XGBoost and LR approaches processed a gradual and nearby result with precision and accuracy values of 92%, 92.12% for the former approach and 92%, 91.57% for the latter approach correspondingly. DTL method exhibited manageable precision and accuracy values of 92.59% and 90.75% while the MLP method exhibited 93% and 93.13% for the same parameters. The proposed FM-HCF-DLF approach accomplished excellent precision and accuracy values, such as 94.85% and 94.08%. The figure represents that the method coined by Xiaowei Xu et al., yielded a least *F* score of 86.70%. Similarly, the DT model accomplished closer *F* score values of 87%. Likewise, the KNN approach implied a moderate *F* score value of 89%. XGBoost and LR technologies achieved same *F* score value of 92%. Simultaneously, the MLP model resulted in a better *F* score value of 93%. The presented FM-HCF-DLF method yielded an optimal *F* score value of 93.20%.

The above-mentioned tables and figures indicate that the FM-HCF-DLF model is an effective classification model compared to other models. The experimental outcomes indicate that the proposed model demonstrated its effective performance by attaining the maximum average sensitivity of 93.61%, specificity of 94.56%, precision of 94.85%, accuracy of 94.08%, *F* score of 93.20% and kappa value of 93.50%. The proposed model accomplished better performance due to the inclusion of fusion-based feature extraction model and Adam optimizer.

## Conclusion

The authors developed an effective FM-HCF-DLF model for COVID-19 diagnosis and classification. The FM-HCF-DLF model involved preprocessing stage using GF technique to remove the noise that exists in the image. Then, the FM-based feature extraction process was performed to extract the useful set of features from the preprocessed image. The HCF features used LBP while the DLF used CNN-based Inception v3 model. Besides, Adam optimizer was applied to adjust the learning rate of Inception v3 model. At last, MLP-based classification process was performed to identify and classify the chest X-ray images into different set of classes. The FM-HCF-DLF model was simulated using chest X-ray dataset which attained the maximum outcome. The respective parameters were maximum sensitivity 93.61%, specificity 94.56%, precision 94.85%, accuracy 94.08%, *F* score 93.2% and kappa value 93.5%. In future, the FM-HCF-DLF model can be improved using other classifiers instead of MLP.

## Appendix 1

Homepage of FM-HCF-DLF model.

## Appendix 2

Training process of FM-HCF-DLF model.

## Appendix 3

Classification process of FM-HCF-DLF model.

## Appendix 4

Classification output of FM-HCF-DLF model.
